# The Use of Antimicrobials in Italian Heavy Pig Fattening Farms

**DOI:** 10.3390/antibiotics9120892

**Published:** 2020-12-10

**Authors:** Federico Scali, Giovanni Santucci, Antonio M. Maisano, Francesca Giudici, Federica Guadagno, Matteo Tonni, Alberto Amicabile, Nicoletta Formenti, Enrico Giacomini, Massimiliano Lazzaro, Giorgio Bontempi, Nicoletta Vitale, Lis Alban, Jeroen Dewulf, Adriana Ianieri, Sergio Ghidini, Giancarlo Belluzzi, Loredana Candela, Angelica Maggio, Paolo Pasquali, Silvio Borrello, Giovanni L. Alborali

**Affiliations:** 1 Istituto Zooprofilattico Sperimentale della Lombardia e dell’Emilia Romagna ‘Bruno Ubertini’ (I.Z.S.L.E.R.), Sector Diagnostic and Animal Health, Via Bianchi 7/9, 25124 Brescia, Italy; giovanni.santucci@izsler.it (G.S.); antoniomarco.maisano@izsler.it (A.M.M.); francesca.giudici@izsler.it (F.G.); federica.guadagno@izsler.it (F.G.); matteo.tonni@izsler.it (M.T.); alberto.amicabile@aulss9.veneto.it (A.A.); nicoletta.formenti@izsler.it (N.F.); giacominienrico81@gmail.com (E.G.); massimiliano.lazzaro@ats-brescia.it (M.L.); giorgio.bontempi@izsler.it (G.B.); giovanni.alborali@izsler.it (G.L.A.); 2 Istituto Zooprofilattico Sperimentale del Piemonte, Liguria e Valle d’Aosta (I.Z.S.T.O.), S.S. Osservatorio delle Regioni, Via Bologna 148, 10154 Turin, Italy; nicoletta.vitale@izsto.it; 3 Danish Agriculture & Food Council, Axelborg, Axeltorv 3, 1609 Copenhagen, Denmark; lia@lf.dk; 4 Veterinary Epidemiology Unit, Faculty of Veterinary Medicine, Ghent University, Salisburylaan 133, 9820 Merelbeke, Belgium; jeroen.dewulf@ugent.be; 5 Department of Food and Drug, Parma University, Via del Taglio 10, 43126 Parma, Italy; adriana.ianieri@unipr.it (A.I.); sergio.ghidini@unipr.it (S.G.); giancarlobelluzzi@gmail.com (G.B.); 6 Italian Ministry of Health, Viale Giorgio Ribotta 5, 00144 Rome, Italy; l.candela@sanita.it (L.C.); a.maggio@sanita.it (A.M.); s.borrello@sanita.it (S.B.); 7 Department of Food Safety Nutrition and Veterinary Public Health, Istituto Superiore di Sanità, Viale Regina Elena 299, 00161 Rome, Italy; paolo.pasquali@iss.it

**Keywords:** AMU, prudent use, mortality, pig production, swine, DDDvet, DDDAit

## Abstract

Data on antimicrobial use (AMU) in heavy pig production (>150 kg) are limited. The aim of this study was to investigate the AMU in this production. Data from 2015 were collected for 143 fattening farms. The AMU was estimated through a treatment index per 100 days (TI_100_) using the defined daily dose animal for Italy (DDDAit). When possible, a comparison with the European Medicines Agency’s defined daily doses for animals (DDDvet) was performed. The median TI_100_ was 10.7 (range, 0.2–49.5). Group treatments represented 94.6% of overall consumption. The AMU calculated using DDDAit and DDDvet were strongly correlated (ρ = 0.976; *p* < 0.001). The AMU was negatively correlated with injectables use (ρ = −0.46, *p* < 0.001) and positively correlated with oral products (ρ = 0.21, *p* = 0.014), premixes (ρ = 0.26, *p* = 0.002), and mortality (ρ = 0.18; *p* = 0.027). Farm size was negatively correlated with AMU (ρ = −0.29, *p* < 0.001). Smaller farms were more frequently above the median TI_100_ (odds ratio = 2.3, 95% confidence interval = 1.2–4.7), suggesting that they may have lower biosecurity and management standards. The results of this study should provide useful insights for the development of an Italian monitoring system.

## 1. Introduction

Antimicrobial use (AMU) in livestock represents an important risk for the selection and spread of antimicrobial resistance (AMR) [1,2], which can severely impair the effectiveness of antimicrobials for human and veterinary medicine. Potential AMR issues because of non-human AMU have been known for more than a decade. In 2007, the World Health Organization (WHO) published the first report on critically important antimicrobials for human medicine [3], which now is at its sixth revision [4], and provided guidance regarding prioritization in preserving the efficacy of antimicrobial classes, particularly the highest priority critically important antimicrobials (HPCIAs). Italy is both an important livestock producer, and in terms of annual sales, one of the highest consumers of antimicrobials in animals within the European Economic Area (EEA) [5]. Italy, with over 11 million pigs slaughtered per year [6], has well-established swine production, and pigs may represent a concerning source of AMR. For instance, an increasing prevalence of multi-resistant *Escherichia coli* was found in Italian pigs between 2002 and 2011 [7]. Methicillin-resistant *Staphylococcus aureus* (MRSA) were identified in more than 35% of pig nasal swabs collected at an Italian slaughterhouse [8], and according to a recent Italian study, the *mcr-1* gene may be common in pathogenic *E. coli* in Italian pigs [9]. Italian pig farming differs from the other EEA countries due to its longer production cycle, which is necessary to rear the typical Italian heavy pigs that are usually slaughtered at 160 kg or more [10]. Several studies on AMU in pig farms are available from various countries [11,12,13,14,15,16,17,18,19,20]. However, the information on AMU in heavy pig production is still limited. The availability of those data at the farm level represents an important step toward the development of an adequate antimicrobial stewardship program [21].

Although different metrics may be used to estimate the AMU at the farm level, international agreement regarding a unique indicator for AMU in animals has still not been obtained [22]. Among the available metrics, the ones based on the concept of “defined daily dose” (DDD) are used in countries such Belgium, Denmark, and the Netherlands in their respective nation-wide monitoring systems [23]. The European Surveillance of Veterinary Antimicrobial Consumption (ESVAC) project of the European Medicines Agency (EMA) developed standardized units of measurements, namely, defined daily doses for animals (DDDvet)—for cattle, pigs, and poultry [24]. 

During the last few years, initiatives were established to address the misuse of antimicrobials in the European Union and in Italy. For instance, in 2016 the European Commission issued a ban on oral veterinary medicinal products (VMPs) that contain colistin in combination with other antimicrobials (Commission Implementing Decision C(2016)4708). The EMA advised countries with a high use of colistin, such as Italy, to reduce their consumption to 5 mg/PCU or less. In Italy, during 2017, a national plan was initiated aiming at a 30% reduction of antimicrobial sales by the end of 2020 [25]. 

The aim of the present study was to provide qualitative and quantitative information on the AMU in Italian heavy pig production, as a starting point before the recently started national initiatives to reduce AMU, through an investigation of fattening farms considering a DDD-based metric.

## 2. Results

**Figure 1:**
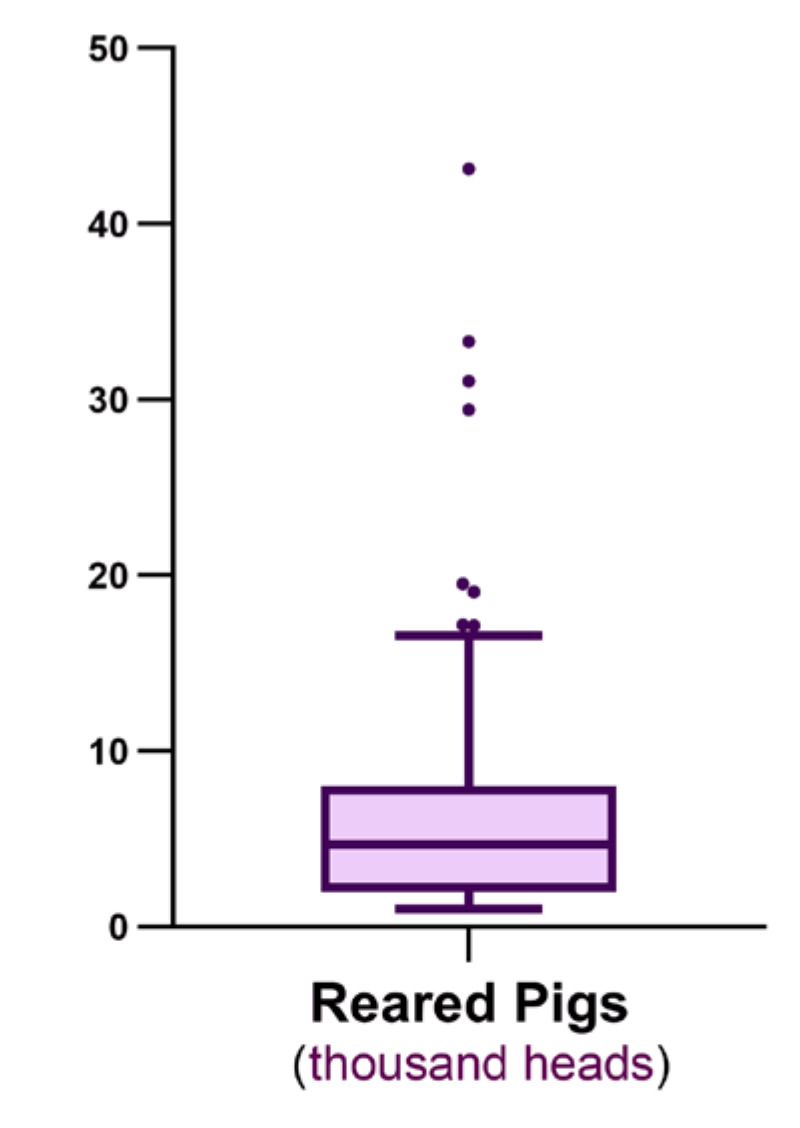
A box and whisker plot (Tukey) showing the distribution of the number of pigs reared (thousand heads) on 143 Italian farms during 2015. The median was 4697 pigs, ranging from a minimum of 1014 to a maximum of 43,159.

A total of 916,276 pigs were reared in the 143 farms during 2015, with a median of 4697 pigs per farm, ranging from 1014 to 43,159 pigs (Figure 1). The median mortality was 4.0% (range, 0.2–12.5%), and median weight at slaughter was 169 kg (range, 137–182 kg).

**Figure 2:**
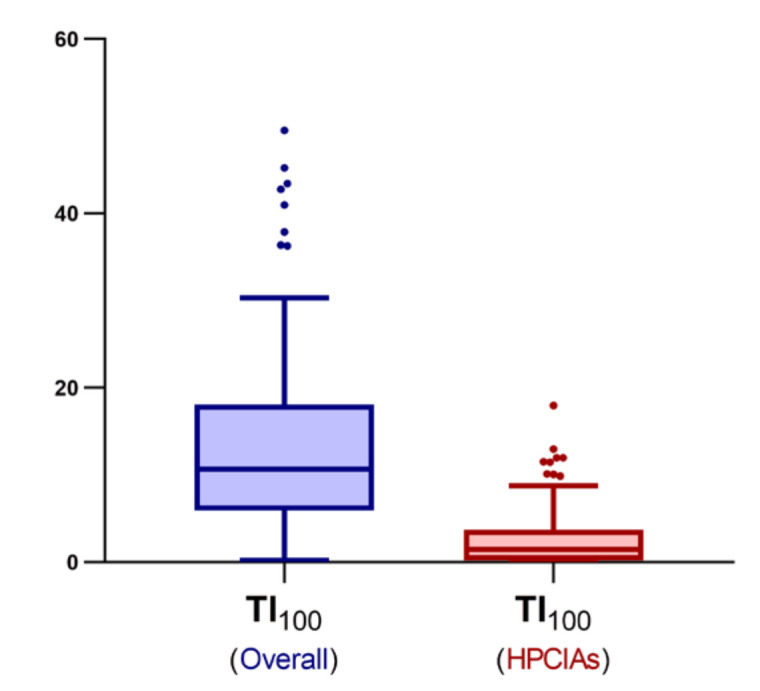
A box and whisker plot (Tukey) showing the distribution of the overall treatment index per 100 days (TI_100_) for 143 Italian pig farms during 2015. The median was 10.7, ranging from a minimum of 0.2 to a maximum of 49.5. The TI_100_ for World Health Organization’s highest priority critically important antimicrobials (HPCIAs) [4] only is also shown. The median was 1.5, ranging from a minimum of 0.0 to a maximum of 18.0.

The median treatment index per 100 days (TI_100_) was 10.7 (range, 0.2–49.5) while the median HPCIAs TI_100_ was 1.5 (range, 0.0–18.0). Figure 2 shows the distribution of the overall TI_100_ and HPCIAs TI_100_.

Overall, HPCIAs were used in 93.7% of the 143 farms, representing 16.8% of the total AMU, and the use was distributed as follows: macrolides 8.8%, polymyxins 5.3%, quinolones 2.6%, and third and fourth generation cephalosporins 0.1%. The five most used classes were tetracyclines (26.6%), lincosamides (22.1%), penicillins (13.4%), pleuromutilins (9.2%), and macrolides (8.8%), representing 80.2% of the total AMU. The use of injectable antimicrobials represented 5.4% of the AMU, and the remaining 94.6% was ascribed to orally administered products. Oral powder and solutions represented 51.7% of the overall AMU, and premixes represented 42.9%. Table 1 shows the distribution of the AMU, divided into classes and formulations, administered in the sampled farms based on the TI. Table 1 also contains the reported percentage of farms that used a given class or formulation at least once during 2015 and the percentages of use (median and range) of those classes and formulations at the farm level.

**Figure 3:**
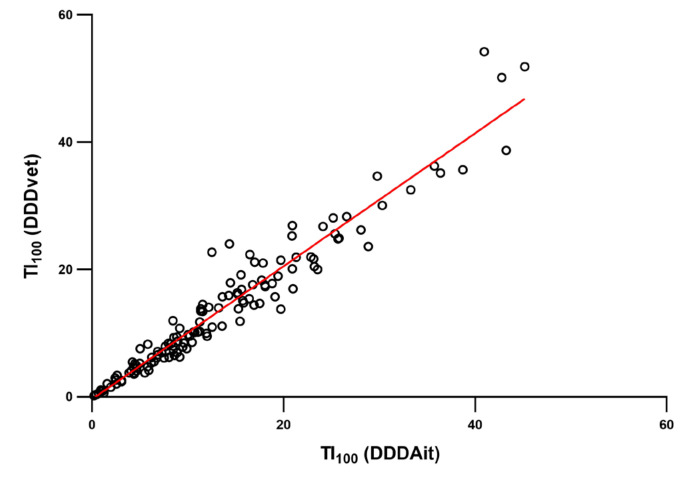
Scatter plot and trend line of 2015 TI_100_ in 143 Italian fattening farms included in this study, where each dot represents a farm. TI_100_ was calculated using either the defined daily dose animal for Italy (DDDAit) or the European Medicine Agency’s defined daily doses for animals (DDDvet) [24] considering only antimicrobials where the DDDvet was available. The two metrics were significantly correlated (ρ = 0.98; *p* < 0.001).

Overall, 94.3% of the AMU quantified using defined daily dose animal for Italy (DDDAit) as a metric could also be measured with DDDvet. Treatment index per 100 days (TI_100_) calculated using DDDAit and DDDvet showed a significant positive correlation (ρ = 0.976; *p* < 0.001). Figure 3 illustrates the relationship between AMU at the farm level estimated using the two different TI_100_.

**Figure 4:**
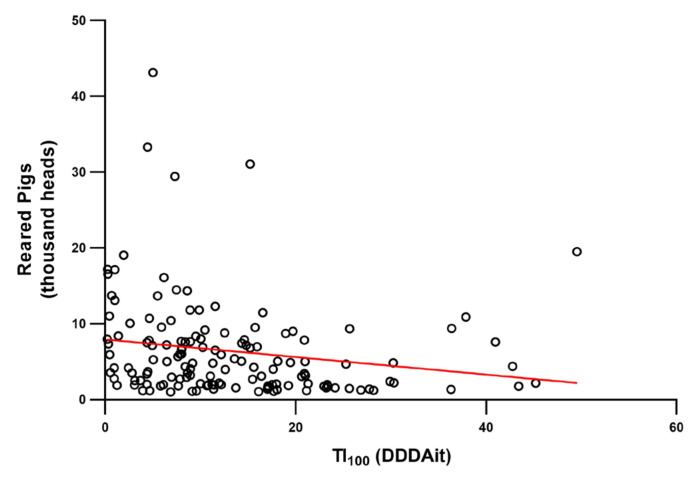
Scatter plot and trend line of 2015 TI_100_ and number of reared pigs in 143 Italian fattening farms included in this study, where each dot represents a farm. A significant negative correlation (ρ = −0.29, *p* < 0.001) was found between TI_100_ and reared pigs.

The use of antimicrobials was negatively correlated with number of reared pigs (ρ = −0.29, *p* < 0.001) and the frequency of injectable use (ρ = −0.46, *p* < 0.001). Figure 4 shows the relationship between AMU and number of reared pigs.

**Figure 5:**
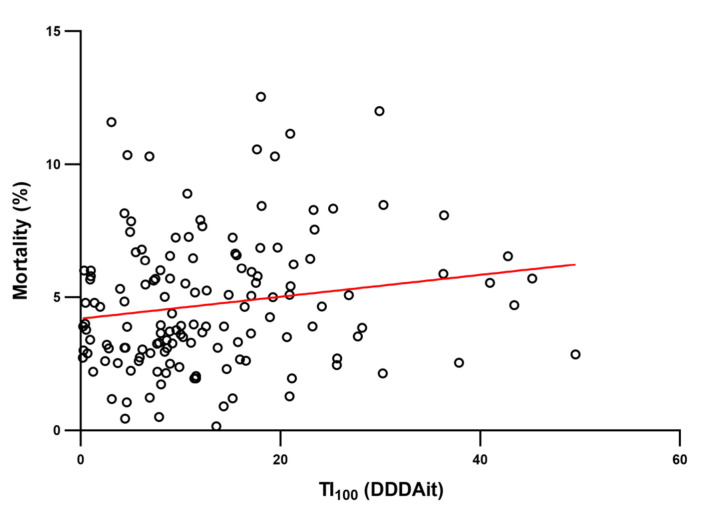
Scatter plot and trend line of 2015 TI_100_ and mortality in 143 Italian fattening farms included in this study, where each dot represents a farm. A significant positive correlation (ρ = 0.18, *p* = 0.027) was found between TI_100_ and mortality.

On the other hand, the AMU was positively correlated with frequency of use of oral products (ρ = 0.21, *p* = 0.014), premixes (ρ = 0.26, *p* = 0.002), and mortality (ρ = 0.18; *p* = 0.027). Figure 5 shows the relationship between AMU and mortality. The number of reared pigs and mortality were negatively correlated (ρ = −0.23, *p* = 0.005).

According to the logistic regression model, a TI_100_ over 10 was found more frequently in farms with less than 5000 pigs reared per year than in larger herds, with an odds ratio (OR) of 2.3 and a 95% confidence interval (95% CI) of 1.2 to 4.7 compared to larger farms. Penicillin was used more frequently in smaller herds (OR = 5.4; 95% CI = 1.1–26.0) compared to larger herds, and more frequently in farms that never used HPCIAs during 2015 (OR = 12.6; 95% CI = 2.8–56.3) compared to herds that did use HPCIAs.

## 3. Discussion

Although limited information on AMU in heavy fattening farms is available, a median TI_100_ of 10.7 should be considered high. For instance, during the same year (2015) in Denmark, the average TI_100_ in fattening farms was around 2 [26]. Even with all the limits of a direct comparison (diverse weights, DDD, etc.), a five-fold difference still underlines how high the AMU was in the investigated farms. AMU is typically higher in younger age groups of animals [23], which suggests that in the early stages of Italian pig production, such as weaning, the AMU is likely even higher. The AMU may be further underestimated due to the 100 kg standard weight. This weight, which should be reviewed in the future, was chosen in 2016, when treatments during the second half of the finishers cycle were common. Since this study was based on paper sources, it was not feasible to verify this assumption. Standard weights may also vary in countries with well-established monitoring systems and similar productions. For example, in Belgium and Denmark, 50 kg is used [27,28], whereas in the Netherlands, it is up to 70 kg [29].

Wide differences in AMU were found among farms, similarly to what has been described in previous studies [11,12,14,19]. In contrast, years of strict restrictions in Denmark have reduced such variability [30]. Those differences should be considered when implementing strategies to reduce AMU, which should start targeting farms with the highest consumption. In addition, farms with lower AMU could be used as positive examples. 

The use of HPCIAs was relatively high (16.7% of total AMU) and frequent (93.7% of the farms). Particularly, macrolides were used in over 60% of farms representing almost 9% of the overall AMU. EMA recently classified macrolides in a category of less importance than other HPCIAs [31]. One of the criteria for considering macrolides as HPCIAs is related to the risk of transmission of resistant *Campylobacter* spp. from non-human sources. Pig farming represents a less relevant source of *Campylobacter* than other production, such as poultry [32,33,34]. 

The use of colistin was also frequent (over 50% of the farms) and relatively high (5.3% of the total AMU), which could be explained by the low awareness of stakeholders in 2015. Indeed, the *mcr* gene was discovered around the end of that year [35], when colistin was still not considered an HPCIA [36]. The sales of colistin in Italy dropped by almost 90% from 2015 to 2018 [5]. Finally, third and fourth generation cephalosporins were rarely used (0.1% of the overall AMU and in less than 10% of the farms), which suggests that it will be feasible to remove those products from fattening pig farms entirely. 

Farms that frequently used injectable VMPs were associated with lower AMU; however, such antimicrobials were rarely administered (5.4% of overall AMU), which highlights the importance of promoting parenteral treatments whenever possible.

Comparing the results of this study with previous ones is difficult due to the lack of a common standard—namely, wide differences in the recommended dosages among countries [37], the estimation of DDD for combination VMPs (with more than one antimicrobial), and standard weights [22]. Using ESVAC’s DDDvet could improve the comparability among studies, but this information is still incomplete. In this study, DDDvet were not available for 7% of the consumed antimicrobials; however, in other scenarios, this gap may be larger. For instance, in a recent study on Italian beef over 25% of the AMU measured using DDDAit came from antimicrobials for which a DDDvet was not available [38]. 

The number of reared pigs, which can be considered as a proxy for herd size, was negatively associated with mortality and AMU. These results may be explained by larger farms paying more attention to biosecurity and management. A positive relationship between farm size and biosecurity was described in a Belgian study [39] and higher or improved biosecurity may lead to lower AMU [30,39,40,41,42]. Therefore, factors that could influence AMU (e.g., management, biosecurity, housing system, alternatives to antimicrobials) should be further investigated in Italian pig farms.

Similarly to the findings of a recent Italian study [43], high AMU did not provide any advantage in terms of mortality, which suggests that other factors could play a more important role. This phenomenon was also observed by Kruse and colleagues (2017) who investigated the impact of the use of vaccines on the AMU in Danish pig herds [28]. 

The main aim of this study was to investigate the AMU in Italian heavy pig production. We selected pigs from 20 kg to slaughter to cover the majority of the life span of the fattening pigs. Only finishers farms were included in the study, because investigating farms that reared a single age group simplified the data collection. Indeed, all information came from paper sources, mostly handwritten, and collecting such data was onerous. The sample selection represented an important limit of this study. Even though the 143 farms reared over 900,000 pigs, their representativeness could be limited because they were not randomly selected. The selection was based on convenience, through contacting farmers that were already involved with Health of the Istituto Zooprofilattico Sperimentale Lombardia Emilia Romagna’s (IZSLER) activities. None of the farmers refused to be part of this investigation, which is in contrast with what has been reported by previous studies in other countries [11,15,39]. However, considering the involvement of the Italian Ministry of Health, farmers may have felt obliged to participate even though enlisting was entirely voluntary. To improve the quality, all processes involving AMU data (collection, sorting, etc.) were double checked. The lack of external validation for such data (e.g., invoices from pharmacies) represents another limitation of this study. A similar problem occurred with pig production and mortality information; indeed, these data were reviewed with the farmer during the visit but no external sources for validation were available. Finally, the data of this study originated from 2015, a year before the beginning of the main initiatives to reduce AMU in Italy. Therefore, future investigations on AMU in the farms included in this study could provide useful insights on the effectiveness of those initiatives.

## 4. Materials and Methods 

### 4.1. Sample Selection and Data Collection

A convenient sample of 143 pig farms was selected from the ones included in the 2016–2017 trial studies during the development of the ClassyFarm system (www.classyfarm.it) by the Italian Ministry of Health. ClassyFarm is an ongoing Italian monitoring system, still under development, which aims to monitor and benchmark farms regarding several key topics, such as AMU, biosecurity, and animal welfare. The sample consisted of fattening farms (finishers, from 20–30 kg to slaughter) that reared at least 1000 pigs per year. The sampled farms were located in four administrative regions in the north of Italy (Piedmont, Lombardy, Emilia-Romagna and Veneto). According the Italian Veterinary Database (www.vetinfo.it), approximately 85% of the pigs present in Italy come from one of those four regions [44]. 

All sampled farms were previously involved in activities of the Sector Diagnostic and Animal Health division of IZSLER, a local authority that operates under the Italian Ministry of Health. The farms were selected based upon their expected willingness to cooperate. Farmers were contacted via phone by an operator, who explained the project aim and the kind of data required, and assured that the farmers’ involvement was anonymous without any repercussions regarding potential high AMU. None of the contacted farmers refused to be involved in the study. All farms were visited between June 2016 and December 2017 by two veterinarians, who scanned all paper prescriptions of 2015. Italian law (D.Lgs. 193/2006 and D.Lgs. 143/2007) requires farmers to keep their prescriptions for at least 5 years. A simple form was sent in advance, either via email or dictated over the phone, to the farmers involved asking them to provide basic production data regarding 2015, such as the number of reared pigs, their mortality, and their weight at slaughter. During a subsequent visit, the production data were reviewed with the farmer.

### 4.2. Estimation of Antimicrobial Use

A treatment index 100 (TI_100_) was calculated to estimate the AMU in the involved farms using the defined daily dose animal for Italy (DDDAit) as a standard metric. A DDDAit was previously established during the development of the ClassyFarm system for each ingredient with antimicrobial activity, contained in the Italian VMPs. In general, a DDDAit of a VMP represents the standard amount of active ingredient, in milligrams, administered per kg of live weight per day (mg/kg/d), according to the summary of the product characteristic (SPC). In the case where a range was expressed for the SPC, the average value was used (e.g., 10 to 20 mg/kg/d, DDDAit = 15). The DDDAit of long-acting active ingredients was established by dividing the dose by the number of days of the therapeutic effect, with an approach similar to the principles on assignment of ESVAC’s DDDvet [45]. The TI_100_ was calculated according to the following formula [21,46]:

TI_100_ = [(Amount of active ingredient used (mg) per farm in 2015) / (DDDAit (mg/kg/d) × animal at risk × weight at risk (kg) × days at risk)] × 100,

where “animals at risk” represents the number of pigs reared during 2015, as declared by the farmer, and “weight at risk” and “days at risk” were standardized at 100 kg and 180 days, respectively.

The TI_100_ is a standard measure of AMU, which can be interpreted in three ways [21]: (1) the percentage of times that any animal was under treatment during its production cycle, (2) the number of days under treatment per 100 days of production, or (3) the number of animals under treatment per 100 animal present in the farm on any given day.

The TI_100_ was calculated for the total AMU and HPCIA use according to WHO’s classification [4]. Namely, cephalosporins (3rd and 4th generation), macrolides, polymyxins, and quinolones (fluoroquinolones and other) were considered HPCIAs. Other HPCIA classes, such as 5th generation cephalosporins and glycopeptides, were not available for veterinary use. The antimicrobial classification proposed by WHO encompasses two levels of prioritization. 

To compare DDDAit with ESVAC’s DDDvet, another TI_100_, based on DDDvet [24], was also calculated replacing DDDAit with DDDvet in the aforementioned formula. However, a DDDvet standard was not available for 11 active ingredients (listed in Table 2) of the VMPs administered during the study. Hence, the AMU could not be fully calculated using only DDDvet.

### 4.3. Data Management and Statistical Analysis

Eight authors, working in two-person teams, were involved in all operations concerning data collection and processing. These teams sorted the prescriptions selecting only the ones dealing with antimicrobials as injectables, premix, or oral administration (powder and solution). Other forms, such as sprays, were not included in the study due to the difficulty in establishing a proper DDD. Finally, the data relevant to the study were included in the ClassyFarm database using an input interface of an HTML5 web-based application. Calculations of the AMU were performed by the ClassyFarm system, and then the data were exported and analyzed using Microsoft Excel (Microsoft Corp., Redmond, WA, USA). 

The farm was considered the experimental unit, and for all statistical analyses, a P-value of 0.05 was used as the cut-off for significance. Data on AMU and pig production were tested with GrapPad Prism 8.0.1 (GraphPad Software, San Diego, CA, USA) for normality using the Anderson–Darling test, D’Agostino and Pearson test, and Shapiro–Wilk test. Relationships between the two TI_100_ values, calculated with DDDAit and DDDvet, were investigated using Spearman’s rank correlation considering only antimicrobials for which both metrics were available (see also Table 2). No further analyses were performed using DDDvet-based TI_100_ due to the impossibility to fully calculate the AMU for all farms (i.e., DDDvet information was not available for certain active ingredients). The associations between the AMU (overall, HPCIAs, and formulation) and production data (reared pigs and mortality) were assessed using Spearman’s rank correlation. All Spearman’s correlations were performed with GrapPad Prism 8.0.1.

Logistic regression was performed using SAS software, version 9.4 (SAS Institute Inc., Cary, NC, USA). The factors TI_100_, number of reared pigs, and mortality were transformed in dichotomic variables using the median value as the cut-off, obtaining respectively, TI_100_ (≤10; >10), pigs (≤5000; >5000 heads), and mortality (≤4%; >4%). For each of those three factors (TI_100_, pigs, and mortality), the associations between them and the variables use of HPCIAs, tetracyclines, penicillins, macrolides, and lincosamides were calculated using the χ2 test by PROC FREQ. 

For each factor (TI_100_, reared pigs, and mortality), all variables with *p* < 0.05 (two-sided) in bivariate analysis were analyzed using three multivariable logistic binomial regression models of fixed effects, using the log–log distribution function of the logit procedure to obtain adjusted odd ratios (OR). The likelihood ratio test was used to assess the overall significance of the three models (two-tailed significance level *p* ≤ 0.05). For each model, the Hosmer–Lemeshow test was performed to assess the model’s goodness-of-fit [47]. Collinearity between the variables was investigated by χ^2^ analysis; in the case of multicollinearity among covariates, a polychoric correlation was used to select the variables.

### 4.4. Data Availability

Due to the sensitive nature of the collected information, the data presented in this study shall be available on reasonable request from the corresponding author with the anonymization of the participating farms and farmers and the VMPs labels.

## 5. Conclusions

In this study, we found relatively high levels of AMU and frequent use of HPCIAs with great differences among the farms investigated. In particular, farms with larger herds were found to administer less antimicrobials compared to farms with smaller herds. Group treatments represented the majority of the consumed antimicrobials. Our results suggest that strategies to reduce AMU at the farm level could start by targeting specifically high consuming farms and promoting individual treatments through improvement of the herd’s health.

## Figures and Tables

**Table 1:** Distribution of the total antimicrobial use (AMU), stratified by class or formulation, for 143 Italian pig farms during 2015. In addition, the percentage of farms that used a given class or form, at least once, is also reported (use> 0) and the percentage of consumption at the farm level (median and range).

Class	Total AMU (%)	% of farms with use> 0	% of AMU at Farm-Level:Median (Range)
Aminocyclitols	0.4	45.5	0 (0–5.4)
Aminoglycosides	3.4	11.2	0 (0–32.6)
Amphenicols	2.2	82.5	1.1 (0–100)
Cephalosporins (III and IV gen.)	0.1	9.8	0 (0–6.7)
Lincosamides	22.1	69.2	3.2 (0–95.8)
Macrolides	8.8	63.6	2.1 (0–82.3)
Penicillins	13.4	91.6	11.9 (0–74.4)
Penicillins (antistaphylococcal)	0.2	51.7	< 0.1 (0–5.8)
Pleuromutilins	9.2	55.9	1.9 (0–89.3)
Polymyxins	5.3	51.7	0.1 (0–55.3)
Quinolones	2.6	58.7	0.1 (0–54.6)
Sulphonamides	5.6	26.6	0 (0–93.3)
Tetracyclines	26.6	83.2	24.5 (0–97.9)
			
**Formulation**			
Injectables	5.4	96.5	3.1 (0–100)
Premixes	42.9	72.7	42.9 (0–99.9)
Oral (powder and solutions)	51.7	85.3	40.2 (0–100)

**Table 2:** List of the active ingredients with their classes, forms of administration, and World Health Organization (WHO) ranking [4] for which DDDvet [24] values were not available.

Active Ingredient	Class	Form
Dicloxacillin	Penicillins (antistaphylococcal)	Injectable
Sulfadiazine	Sulphonamides	Injectable
Sulfadimidine	Sulphonamides	Injectable
Sulfamerazine	Sulphonamides	Injectable
Tildipirosin	Macrolides	Injectable
Tulathromycin	Macrolides	Injectable
Ampicillin	Penicillins	Premix
Flumequine	Quinolones	Premix
Gentamicin	Aminoglycosides	Premix
Sulfamerazine	Sulphonamides	Premix
Thiamphenicol	Amphenicols	Premix
Dicloxacillin	Penicillins (antistaphylococcal)	Injectable
Sulfadiazine	Sulphonamides	Injectable
Sulfadimidine	Sulphonamides	Injectable
Sulfamerazine	Sulphonamides	Injectable
